# Northeast network in biotechnology (RENORBIO)

**DOI:** 10.1186/1753-6561-8-S4-O44

**Published:** 2014-10-01

**Authors:** Maria Madalena Guerra, Aurea Wischral, Paula Lenz, Mitermayer Reis

**Affiliations:** 1 Federal Rural University of Pernambuco, Pernambuco, Brazil; 2 State University of Ceará, Fortaleza, Brazil; 3 Oswaldo Cruz Foundation, Rio de Janeiro, Brazil

## 

The Northeast Network in Biotechnology (RENORBIO) was established in 2004 by the Federal Government that identified the demand for this type of professional and signalizes a great opportunities them to act on the growing industry of biotechnology in Brazil. It is a network of research, development and innovation, which base is the Post-Graduation Program (PGP-RENORBIO) that have 36 institutions of education and research in the nine states of the Northeast, besides Espírito Santo, which also integrates this initiative. Started in September 2006, it represents a new model of Post-graduate program - a network program - fully supported by CAPES direction.

The PGP-RENORBIO specifically aims to qualifying researchers, in a doctorate level, with a solid technical-scientific basis, able to act on several markets, such as teaching, research, services and industry. This way their specific objectives are: 1) Training qualified people for the activity of research and of higher teaching in the field of Biotechnology; 2) Encourage research in the area of Biotechnology, under the multi e interdisciplinary perspective; 3) Producing, expanding and applying knowledge to Biotechnology to the economical and cultural reality of the Northeast region.

In the long run, it is hoped that the PGP-RENORBIO, as it make possible the interaction of institutions of research and teaching, national and international, and the best use of the critical mass existing in the Northeast region, contribute to the process of consolidation of the Northeast Biotechnology Network and to the effective to and systematic development of Biotechnology in the Country.

The RENORBIO's PGP assumes that it is possible to obtain growth with quality through means of integration strategies which promote development with Competitiveness, according to a world tendency. Hence, the integration of consolidated and emergent groups constitutes the basis of the strategy of the RENORBIO Program. Take part in this effort about two hundred Doctors connected to the thirty-six existing institutions in the nine states of the Northeast, besides Espírito Santo. This is being made possible because of an explicit institutional support, in a form of an Institution's Consortium. Besides supporting the strengthening of the existing programs in the institutions that integrate this initiative, by the input of additional resources to them, this strategy discourages the disordered creation of new programs in Biotechnology in Region, avoiding the lavishness of resources and competencies. On the other hand, taking in account that the program works by adhesion, according to rules of defined institutional association in its regiment, allows the participation of a great number of national and international institutions, such as EMBRAPA and FIOCRUZ, which generally stay aside from the human resources formation efforts in the Country, considering they do not constitute themselves as Institutions of Higher Learning. By means of that integration strategy, all participating institutions, even those of less developed states, may offer a broad résumé academic curriculum related to four areas of concentration where Biotechnology offers its most important applications: Health, Agriculture and Animal Raising, Natural Resources and Industrial Biotechnology.

Science, technology and innovation are essential factors for the development and competitiveness of nations. Countries without scientific competency cannot promote their development based on their own technologies and innovations have to pay to use the innovations developed in other countries. For the last twenty years, Brazil has quadrupled its scientific contribution in the global context. It is notorious this fact that results in the consolidation on the Post-Graduation in the Country, under the leadership of CAPES, since the scientific production and the post-graduation have a direct relation. However, enhance the Brazilian scientific contribution on the international context it is still necessary and, in this sense, it is opportune to stimulate the participation of young scientists in the Brazilian post-graduation system.

Also, to stimulate the participation and insertion of Brazil in the use of the advances of bioscience in order to reduce hunger and minimize the serious problems in public health, especially those related to childhood mortality, it is more than just an opportunity, it is a mission of science. Such advances cannot prescind Biotechnology, a relatively young branch of bioscience, whose full development depends on the elucidation of the main dogmas of biology, which demand high competence and excellence.

Brazil is one of the largest detainers of biodiversity. Northeast Region alone, holds 42% of its area constituted by semi-arid; a region with unique climate and biodiversity in the whole world. That and other characteristics make the scenario for Biotechnology very promising. The progress in this area may be accelerated if there are joined efforts conjugated among government, scientific and corporative community and in the development of joint projects, in the formation of productive partnerships, in the training of human resources, in the creation of a favorable environment to new investments and in the development and/or adaptation of technologies with the objective of broadening Competitiveness and making the market of biotechnological products more dynamic.

The opportunities for alliances and new partnerships between the knowledge generating sector and the goods and services one, represented predominantly by small and medium size companies, constitute focal point of the Biotechnology and Genetic Resources Program, from the Ministry of Science and Technology, which adopts among its strategies of action and organization of network projects to enhance the flow of innovation and channel the production and the commercialization of its results for the benefit of society.

In the long run, it is hoped that RENORBIO constitutes itself into a Center of Excellence in Biotechnology, which will internalize and develop as more advanced technologies for a wide application in all areas of Biotechnology, with adequate levels of excellence and relevance.

Considered a successful program, the PGP-RENORBIO had its General Coordination in the State University of Ceará (UECE) during the period from 2006 to 2012 and now its Coordination is in the Federal Rural University of Pernambuco (UFRPE). Currently PGP-RENORBIO has 246 professors and 520 students; oversees 15 post-doctorates, and has trained 281 doctors and completed the supervision of eleven postdocs. The results obtained with the development of the student research made possible to, in five years, increase the number of patent applications from 8 to 216, the number of professors involved in patent rose from 6 to 70; addition to the considerable increase on the production of papers from its researchers and students, having also been a significant increase in publications in journals with high impact factor. For a biotechnology program so young, these data are of great relevance, showing that there is a substantial change in the culture of generation of products / processes in the region.

So, the concern of PGP-RENORBIO is the transfer, effectively, of the processes and innovative products generated in their laboratories in order to produce wealth, life quality and social inclusion in the Northeast Region. This challenge gets to be conquered by the creation of the first 11 companies from students, graduates and professors, as well as the licensing of 17 inventions and 17 other negotiations. Thus, PGP-RENORBIO has established close collaboration with industry and confirming your main goal to train qualified personnel in close relation to the needs of the productive sector.

